# Direct and Inverse Steady-State Heat Conduction in Materials with Discontinuous Thermal Conductivity: Hybrid Difference/Meshless Monte Carlo Approaches

**DOI:** 10.3390/ma18184358

**Published:** 2025-09-18

**Authors:** Sławomir Milewski

**Affiliations:** Faculty of Civil Engineering, Cracow University of Technology, 31-155 Cracow, Poland; slawomir.milewski@pk.edu.pl

**Keywords:** steady-state heat conduction, discontinuous thermal conductivity, direct and inverse problems, Monte Carlo method, meshless method, numerical analysis

## Abstract

This study investigates steady-state heat conduction in materials with stepwise discontinuities in thermal conductivity, a phenomenon frequently encountered in layered composites, thermal barrier coatings, and electronic packaging. The problem is formulated for a 2D two-domain region, where each subdomain has a distinct constant conductivity. Both the direct problem—determining the temperature field from known conductivities—and the inverse problem—identifying conductivities and the internal heat source from limited temperature measurements—are addressed. To this end, three deterministic finite-difference-type models are developed: two for the standard formulation and one for a meshless formulation based on Moving Least Squares (MLS), all derived within a local framework that efficiently enforces interface conditions. In addition, two Monte Carlo models are proposed—one for the standard and one for the meshless setting—providing pointwise estimates of the solution without requiring computation over the entire domain. Finally, an algorithm for solving inverse problems is introduced, enabling the reconstruction of material parameters and internal sources. The performance of the proposed approaches is assessed through 2D benchmark problems of varying geometric complexity, including both structured grids and irregular node clouds. The numerical experiments cover convergence studies, sensitivity of inverse reconstructions to measurement noise and input parameters, and evaluations of robustness across different conductivity contrasts. The results confirm that the hybrid difference-meshless Monte Carlo framework delivers accurate temperature predictions and reliable inverse identification, highlighting its potential for engineering applications in thermal design optimization, material characterization, and failure analysis.

## 1. Introduction

Materials with discontinuous thermal properties are frequently encountered in various engineering applications. A sharp change, or stepwise jump, in thermal conductivity across an interface occurs naturally in many layered or composite systems [1]. Common examples include sandwich panels with metallic facings [2] and low-conductivity polymer or foam cores [3], metal–ceramic composites designed for high-temperature environments [4], and thermal barrier coatings (TBCs) used in aerospace turbine components [5]. In electronics, abrupt conductivity changes appear at metal–semiconductor interfaces (e.g., copper–silicon) [6] or between ceramic substrates and metallic traces in power modules [7]. In civil engineering, thermal conductivity discontinuities are present in multilayer walls, such as brick–insulation systems [8,9], and in reinforced concrete structures [10], where steel reinforcement has much higher thermal conductivity than the surrounding concrete.

Recent studies have highlighted the importance of modeling heat conduction in materials with heterogeneous or layered structures. Ganguly et al. [11] investigated thin photopolymerized coatings with distinct thermal properties, demonstrating practical applications involving localized heating, while He et al. [12] applied physics-informed deep learning to solve direct and inverse heat conduction problems in complex materials. Accurately capturing variations in material properties is crucial due to the fact that at the interface between two media, the temperature field remains continuous, whereas the heat flux—governed by Fourier’s law—changes according to the conductivity ratio, resulting in a discontinuity in the temperature gradient [13,14,15]. Such discontinuities have direct practical implications for thermal design, energy efficiency, and failure analysis, particularly in high-gradient environments or when precise temperature predictions are required [16,17].

From an application standpoint, accurately capturing these discontinuities enables the development of computational tools that can predict temperature fields and heat fluxes in engineering components with high fidelity. This includes optimizing thermal barrier coatings in turbines to prevent overheating [18], designing composite walls in buildings for improved energy efficiency [19], and predicting hot spots in electronic devices to avoid failure [20]. The numerical methods presented in this work, though derived from mathematical formulations, are intended to provide direct, physically meaningful estimates of temperatures and fluxes in real-world components, bridging the gap between mathematical modeling and practical engineering analysis. These considerations underline the relevance of the study for engineers and designers working with heterogeneous or layered materials.

From a modeling perspective, discontinuous coefficients introduce mathematical and numerical challenges. The presence of a sharp interface complicates the solution of governing equations, as it requires the correct enforcement of interface conditions and can lead to local singularities in the derivative fields [21,22]. Numerical methods that handle such discontinuities efficiently are therefore essential for obtaining accurate and stable solutions, both in direct analyses and in inverse problems such as thermal property identification [23,24] or reconstruction of the heat source term [25,26].

While the finite element method (FEM), with its numerous extensions and couplings with other approaches, remains the most widely used numerical tool for solving steady-state heat conduction problems [27,28,29], its application to media with discontinuous thermal conductivity is still fraught with challenges. In the standard FEM framework, the governing equations are reformulated in a variational form that involves integrals of continuous operators over the computational domain. When the heat flux field is discontinuous due to abrupt conductivity changes, accurate numerical integration across interfaces becomes non-trivial. Additionally, the FEM requires mesh generation, often with refinement near material boundaries, and the resulting temperature field is expressed as a global approximation with a prescribed degree of continuity. This inherent smoothness may hinder the accurate representation of sharp variations in temperature gradients at material interfaces.

Several studies have applied the FEM and its variants to problems with discontinuous thermal properties, demonstrating the method’s versatility but also highlighting practical limitations. For instance, specialized interface elements [30] or adaptive mesh refinement strategies [31] are often necessary to capture the temperature gradient jumps accurately. Such requirements increase computational cost and can complicate the implementation [32], particularly for inverse problems or optimization tasks [33]. By contrast, the proposed hybrid difference/meshless Monte Carlo approaches provide an alternative framework that directly addresses interface discontinuities, allowing for localized enforcement of conditions and efficient point-wise estimation of temperatures, which can simplify practical engineering analyses. Moreover, this Monte Carlo formulation can be seen as an attempt to create a digital twin of a material sample with discontinuous thermal conductivity, on which laboratory-like experiments can be simulated. In such experiments, measurements are typically taken at selected points rather than across the entire sample surface, a scenario that the Monte Carlo model naturally accommodates, both for direct problems and for inverse identification tasks.

In this work, we employ a local formulation of the heat conduction problem combined with numerical approaches that enforce only local continuity of the solution within the neighborhood of each computational point. This framework allows for more precise control over material discontinuities by shifting the approximation from point to point without performing subdomain integrals, thus avoiding the undesirable over-smoothing effect of the conductivity jump. Two variants are considered: a standard finite difference scheme [34,35], suitable for geometrically simple domains, and a meshless Moving Least Squares (MLS)-based approach [36,37], which can flexibly handle irregular geometries without mesh generation.

Among the numerous applications of mesh-free methods in contemporary physics, many studies focus on elliptic problems in which the solution is assumed to be discontinuous. In [38], a meshless generalized finite difference method was proposed for the Poisson problem with a jump in the diffusion coefficient, employing a conservative formulation based on Voronoi polygons. A similar problem was addressed in [39] using Pascal polynomials as basis functions together with a multiscale approach to obtain numerically stable solutions. The partial differential equation describing strongly nonlinear equilibrium radiation diffusion, with material properties varying between subdomains, is analyzed in [40]. Other noteworthy approaches to problems with material discontinuities include, for instance, the decomposition of an appropriately rescaled multivariate Vandermonde matrix with partial pivoting [41], conversion into two coupled subproblems [42], and enrichment of the standard MLS approximation with additional step, wedge, and scissors functions [43].

Furthermore, the deterministic algorithms developed for standard and meshless FD methods may serve as the basis for a stochastic Monte Carlo approach [44] using the random walk technique [45,46,47]. This enables direct estimation of temperature at any selected point in the domain without computing the entire field. Such a feature is particularly advantageous in inverse problems, where only a limited number of measurement points is available [25,48]. In these cases, the temperature at measurement locations—known from experimental data—can be used to determine unknown parameters in the governing equations throughout the optimization process, including material conductivities and internal heat sources, without the need for full-domain simulations and applications of time-consuming and computationally demanding numerical optimization approaches.

The main objective of this study is to develop, implement, and compare deterministic and stochastic numerical strategies for steady-state heat conduction problems in materials with stepwise discontinuous thermal conductivity, addressing both direct and inverse formulations. Applying local formulations and methods with inherently local continuity, the proposed approach aims to improve the accuracy of interface modeling without the drawbacks of mesh generation, subdomain integration, or excessive solution smoothing. The novelty of this work lies in combining reconstructed local formulation, standard finite difference and meshless MLS-based schemes with a random walk Monte Carlo formulation for interface problems, enabling targeted evaluation of temperature fields in arbitrary points of interest. Such a combination provides computational efficiency in inverse analyses, where only sparse measurement data are available, and where accurate parameter identification or internal heat source reconstruction is required.

The paper is organized as follows. Section 2 formulates the physical problem and presents the local interface conditions for discontinuous thermal conductivity and describes the finite difference, MLS, and Monte Carlo algorithms in detail. Section 3 reports the results of numerical experiments for both direct and inverse problems, whereas their discussion, including performance comparisons and sensitivity analyses, is provided in Section 4. Finally, Section 5 summarizes the findings and outlines future research directions.

## 2. Materials and Methods

In this section, two benchmark steady-state 2D heat conduction problems are analyzed, both involving samples composed of materials with discontinuous thermal conductivity. The first problem is a preliminary example defined on a rectangular domain, where the material interface is a straight line. For this case, two alternative local formulations are derived, leading to two distinct numerical models based on the standard finite difference method with a regular grid and central difference operators. These models are then further extended to their stochastic counterparts following the Monte Carlo random walk framework. The second problem considers a domain with a more complex geometry and an arbitrarily shaped material interface. For this case, a mesh-free finite difference scheme with Moving Least Squares (MLS) approximation is developed, along with its stochastic analog.

### 2.1. Laplace Problem Formulation

The local formulation of the following two-dimensional boundary value problem is considered. We study the second-order elliptic partial differential equation

(1) $$\mathrm{div}\left(\eta (x)\;\;\;\nabla \Phi (x)\right)=0,\;\;\;x\in \Omega =\left(0,1\right)\times \left(0,1\right),$$

subject to the essential (Dirichlet) boundary condition

(2) $$\Phi \left(x\right)=\Phi_{D}\left(x\right),\;\;\;x\in \partial \Omega ,$$

where $x=(x_{1},x_{2})$ denotes the spatial coordinates.

The material coefficient η(x) is assumed to be piecewise constant and strictly positive, with a discontinuity across a vertical interface at $x_{1}=x_{c}$, namely

(3) $$\eta \left(x\right)=\left\{\begin{array}{cc} \eta_{1}, & x_{1}<x_{c},\\ \eta_{2}, & x_{1}>x_{c}, \end{array}\right.\;\;\;x_{c}\in \left(0,1\right).$$

For well-posedness, it is required that $\eta_{1},\;\;\;\eta_{2}>0$ and $\bar{\Phi }$ is sufficiently smooth on ∂Ω. The solution Φ is continuous across the interface $x_{1}=x_{c}$, while the normal heat flux satisfies the transmission condition

(4) $$\eta \frac{\partial \Phi }{\partial x_{1}}=0\;\;\;\mathrm{at}\;\;\;x_{1}=x_{c},$$

ensuring conservation of energy across the material discontinuity.

From the physical point of view, Equation (1) corresponds to the stationary heat conduction problem in a two-layered medium. The field $\Phi \in C^{2}\left(\Omega_{1}\right)\cap C^{2}\left(\Omega_{2}\right),\;\;\;\Phi \in C^{0}\left(\Omega \right)$, where

$$\Omega_{1}=\left\{x\in \Omega :x_{1}<x_{c}\right\},\;\;\;\Omega_{2}=\left\{x\in \Omega :x_{1}>x_{c}\right\}.$$

represents the temperature distribution, while η denotes the thermal conductivity of the respective material region. Condition (2) prescribes the boundary temperature, and the interface relations enforce continuity of temperature and conservation of the normal component of the heat flux across the material interface.

This simplified rectangular setup with a straight material interface serves as a preliminary example, since it allows for a clear identification of the essential features of discontinuous coefficients and provides a controlled benchmark against which more advanced numerical formulations can later be validated. On the other hand, though simple and mathematically well posed, this formulation is not particularly convenient for constructing a numerical model. Therefore, two alternative local formulations, suitable for numerical discretization, will be presented in the following two subsections.

#### 2.1.1. First Continuous Model (CM1)

The differential Equation (1) can be rewritten as

(5) $$\nabla \eta \cdot \nabla \Phi +\eta \nabla^{2}\Phi =0,$$

or, in index notation,

(6) $$\frac{\partial \eta }{\partial x_{1}}\frac{\partial \Phi }{\partial x_{1}}+\frac{\partial \eta }{\partial x_{2}}\frac{\partial \Phi }{\partial x_{2}}+\eta \left(\frac{\partial^{2}\Phi }{\partial x_{1}^{2}}+\frac{\partial^{2}\Phi }{\partial x_{2}^{2}}\right)=0.$$

Since η is discontinuous, the equation can be decomposed over the two subdomains and their interface, namely

(7) $$\left\{\begin{array}{c} \eta_{1}\left(\frac{\partial^{2}\Phi }{\partial x_{1}^{2}}+\frac{\partial^{2}\Phi }{\partial x_{2}^{2}}\right)=0,\;\;\;x_{1}<x_{c},\\ \left(\eta_{2}-\eta_{1}\right)\delta \left(x_{1}-x_{c}\right)\frac{\partial \Phi }{\partial x_{1}}+\frac{\eta_{1}+\eta_{2}}{2}\left(\frac{\partial^{2}\Phi }{\partial x_{1}^{2}}+\frac{\partial^{2}\Phi }{\partial x_{2}^{2}}\right)=0,\;\;\;x_{1}=x_{c},\\ \eta_{2}\left(\frac{\partial^{2}\Phi }{\partial x_{1}^{2}}+\frac{\partial^{2}\Phi }{\partial x_{2}^{2}}\right)=0,\;\;\;x_{1}>x_{c}, \end{array}\right.$$

where distribution derivative $\frac{\partial \eta }{\partial x_{1}}$ is replaced by the one-dimensional Dirac delta function δ, while $\frac{\partial \eta }{\partial x_{2}}=0$ due to the constancy of the material parameter along the $x_{2}$ direction. The system (7) is to be complemented with the boundary conditions from (2). This decomposition highlights the physical interpretation of the model: in each subdomain, the Laplacian term dominates, reflecting diffusion (or conduction) within a homogeneous material, whereas at the interface, the Dirac delta term accounts for the jump in the material parameter, representing a localized discontinuity in heat flux across $x_{1}=x_{c}$.

#### 2.1.2. Second Continuous Model (CM2)

Instead of differentiating the discontinuous material parameter in (1) and the explicit use of distributions, the governing equation may be reformulated by decomposing the problem into two subproblems, each defined on one part of the domain ($\Omega =\Omega_{1}\cup \Omega_{2}$). The two subdomains are coupled by interface conditions, which enforce continuity of the potential Φ and of the normal flux in the $x_{1}$ direction along the common boundary $\partial \Omega_{1,2}=\partial \Omega_{1}\cap \partial \Omega_{2}$. The resulting system of equations takes the form

(8) $$\left\{\begin{array}{c} \eta_{1}\nabla^{2}\Phi =0,\;\;\;x\in \Omega_{1}\\ \eta_{2}\nabla^{2}\Phi =0,\;\;\;x\in \Omega_{2}\\ {\left.\Phi \right|}_{\partial \Omega_{1}}={\left.\Phi \right|}_{\partial \Omega_{2}},\;\;\;x\in \partial \Omega_{1,2}\\ {\left.\eta_{1}\frac{\partial \Phi }{\partial x_{1}}\right|}_{\partial \Omega_{1}}={\left.\eta_{2}\frac{\partial \Phi }{\partial x_{1}}\right|}_{\partial \Omega_{2}},\;\;\;x\in \partial \Omega_{1,2} \end{array}\right.$$

Equation (8) must be supplemented with the external boundary conditions specified in (2).

Physically, this formulation reflects the natural partitioning of the medium into two distinct phases. Each subdomain $\Omega_{1}$ and $\Omega_{2}$ is governed by a homogeneous Laplace equation with its own material coefficient $\eta_{i}$, while the interface conditions ensure the conservation of potential and flux across the boundary $\partial \Omega_{1,2}$. This corresponds directly to continuity of temperature and heat flux in thermal problems or to continuity of electric potential and current density in conduction problems. Although this description is conceptually clear and physically transparent, it is less convenient for direct numerical implementation, which motivates the alternative approaches discussed in the following sections.

### 2.2. Finite Difference Method (FDM)

It is assumed that the computational domain is discretized with a uniform grid $\Omega_{h}$ consisting of N=n×n nodes and its discrete boundary $\partial \Omega_{h}$. The mesh size is defined as the modulus

$$h=\frac{1}{n-1}.$$

Taking advantage of the regular structure of the mesh, continuous differential operators can be replaced by central difference approximations at the internal nodes. For the first derivative, one obtains

(9) $${\left.\frac{\partial \Phi }{\partial x_{1}}\right|}_{\left(i,j\right)}\approx \frac{\Phi_{i+1,j}-\Phi_{i-1,j}}{2h},\;\;\;i,j=2,3,\ldots ,n-1,$$

while the discrete Laplacian is approximated by the well-known five-point stencil

(10) $${\left.\left(\frac{\partial^{2}\Phi }{\partial x_{1}^{2}}+\frac{\partial^{2}\Phi }{\partial x_{2}^{2}}\right)\right|}_{\left(i,j\right)}\approx \frac{\Phi_{i-1,j}+\Phi_{i,j+1}+\Phi_{i+1,j}+\Phi_{i,j-1}-4\Phi_{i,j}}{h^{2}}.$$

For nodes located on the boundary or on an internal interface, central differences cannot always be applied directly. In such cases, the first derivative is approximated using forward or backward difference operators, namely

(11) $${\left.\frac{\partial \Phi }{\partial x_{1}}\right|}_{\left(i,j\right)}\approx \frac{\Phi_{i+1,j}-\Phi_{i,j}}{h}=\frac{\Phi_{i,j}-\Phi_{i-1,j}}{h},\;\;\;i=1,n,\;\;\;j=2,3,\ldots ,n-1.$$

It is worth noting that the central difference schemes (9) and (10) are second-order accurate with respect to the mesh size *h*, which makes them a classical and widely used tool in the finite difference method. In contrast, the forward and backward approximations (11) are only first-order accurate, yet they remain necessary in the vicinity of the boundaries or interfaces.

#### 2.2.1. First Finite Difference Model (FDM1)

We employ the problem formulation introduced in (7). The Dirac delta δ can be approximated by the following analytical function:

(12) $$\delta \left(x-x_{c}\right)\approx \delta_{a}\left(x-x_{c}\right)=\frac{1}{\left|a\right|\sqrt{\pi }}\exp\left[-{\left(\frac{x-x_{c}}{a}\right)}^{2}\right],$$

where the parameter *a* determines the effective support size of the approximation. The Dirac delta can then be interpreted as the limit of $\delta_{a}$ as a→0.

With the use of $\delta_{a}$, the governing differential equation valid at $x_{1}=x_{c}$ may be extended to the entire computational domain in the form

(13) $$\left(\eta_{2}-\eta_{1}\right)\delta_{a}\left(x-x_{c}\right)\frac{\partial \Phi }{\partial x_{1}}+\left(\eta_{1}+\left(\eta_{2}-\eta_{1}\right)H\left(x-x_{c}\right)\right)\left(\frac{\partial^{2}\Phi }{\partial x_{1}^{2}}+\frac{\partial^{2}\Phi }{\partial x_{2}^{2}}\right)=0,$$

where *H* denotes the Heaviside step function, defined as

(14) $$H\left(x-x_{c}\right)=\left\{\begin{array}{cc} 0, & x<x_{c},\\ \frac{1}{2}, & x=x_{c},\\ 1, & x>x_{c}. \end{array}\right.$$

Applying the difference operators (9) and (10), together with the collocation technique, to the above differential equation at the internal and boundary nodes $x_{\left(i,j\right)}$ yields the following algebraic system:

(15) $$\begin{array}{c} \left(\eta_{2}-\eta_{1}\right)\delta_{a}\left(x_{1(i,j)}-x_{c}\right)\frac{\Phi_{i+1,j}-\Phi_{i-1,j}}{2h}\\ +\left(\eta_{1}+\left(\eta_{2}-\eta_{1}\right)H\left(x_{1(i,j)}-x_{c}\right)\right)\frac{\Phi_{i-1,j}+\Phi_{i,j+1}+\Phi_{i+1,j}+\Phi_{i,j-1}-4\Phi_{i,j}}{h^{2}}=0,\;\;\;x_{i,j}\in \Omega_{h}, \end{array}$$

together with the boundary condition

(16) $$\Phi \left(x_{\left(i,j\right)}\right)=\Phi_{D}\left(x_{\left(i,j\right)}\right),\;\;\;x_{i,j}\in \partial \Omega_{h}.$$

#### 2.2.2. Second Finite Difference Model (FDM2)

We now consider the problem formulation given in (8). Since the computational domain is decomposed into two subdomains, independent meshes $\Omega_{1,h}$ and $\Omega_{2,h}$ must be generated in each of them, with overlapping nodes placed along the interface. Consequently, the total number of nodes becomes n×n+n, while the number of unknowns exceeds that of the first model by 2n−4. In this case, second-order central difference operators (10) are applied to internal nodes, whereas first-order forward and backward operators (11) are employed at the interface nodes. The resulting system of algebraic equations can be expressed as

(17) $$\left\{\begin{array}{c} \eta_{1}\frac{\Phi_{i-1,j}+\Phi_{i,j+1}+\Phi_{i+1,j}+\Phi_{i,j-1}-4\Phi_{i,j}}{h^{2}}=0,\;\;\;x_{i,j}\in \Omega_{1,h}\\ \eta_{2}\frac{\Phi_{k-1,l}+\Phi_{k,l+1}+\Phi_{k+1,l}+\Phi_{k,l-1}-4\Phi_{k,l}}{h^{2}}=0,\;\;\;x_{k,l}\in \Omega_{2,h}\\ \Phi_{i,j}-\Phi_{k,l}=0,\;\;\;x_{i,j},x_{k,l}\in \partial \Omega_{1,2}\\ \eta_{1}\frac{\Phi_{i,j}-\Phi_{i-1,j}}{h}-\eta_{2}\frac{\Phi_{k+1,l}-\Phi_{k,l}}{h}=0,\;\;\;x_{i,j},x_{k,l}\in \partial \Omega_{1,2} \end{array}\right.$$

where $i,k=2,3,\ldots ,\frac{n}{2}-1$ and j,l=2,3,…,n−1. The boundary condition in the discrete form remains the same as in (16).

##### Summary of the Two Finite Difference Models

The first finite difference model (FDM1) avoids the need to construct two separate meshes consistent across the interface, which makes its implementation relatively straightforward. However, its accuracy may depend on the choice of the smoothing parameter *a* used in the approximation of the Dirac delta function.

The second model (FDM2), in contrast, requires generating two meshes in the two subdomains, enforcing their consistency along the interface, and introducing additional equations at the interface nodes. This procedure can be technically challenging, especially for domains of arbitrary geometry, even though it directly incorporates the continuity conditions across the interface.

In summary, FDM1 is often more practical from a numerical standpoint, as it simplifies implementation and mesh generation, albeit at the cost of introducing additional distribution approximation. FDM2, while more demanding computationally, can be viewed as more mathematically elegant and accurate in enforcing interface conditions.

### 2.3. Monte Carlo with Random Walk

The Monte Carlo method with random walk provides a probabilistic approach for estimating the solution of the Laplace boundary value problem at a specified interior point of the domain. Its general principles can be summarized as follows:

1. Select a point inside the domain, coinciding with an internal node $x_{i,j}\in \Omega_{h}$ of the regular mesh, and initialize a random walk.
2. Determine the possible move directions and their associated probabilities on the basis of the finite difference equation generated at this node using the collocation technique.
3. Randomly select the next node according to these probabilities.
4. Repeat steps 2–3 until the random walk reaches a boundary node $x_{k,l}\in \partial \Omega_{h}$, where the solution value $\bar{\Phi }\left(x_{k,l}\right)$ is prescribed. At this stage, the random walk is terminated and the corresponding nodal indication counter ${\bar{N}}_{\left(i,j),(k,l\right)}$ for this boundary node is increased by one.
5. Return to the initially selected interior point and repeat steps 2–4 until a total of *N* random walks have been completed.
6. Estimate the solution at the chosen interior point using the Monte Carlo formula
  (18) $$\Phi_{i,j}\approx {\hat{\Phi }}_{i,j}=\frac{1}{N}\sum_{k,l}{\bar{N}}_{\left(i,j),(k,l\right)}\bar{\Phi }\left(x_{k,l}\right).$$

The Monte Carlo estimate (18) converges to the finite difference solution obtained either by (15) or (17), provided that the number of random walks *N* is sufficiently large. Moreover, the Monte Carlo error

(19) $$e=\left|\frac{\Phi_{i,j}-{\hat{\Phi }}_{i,j}}{\Phi_{i,j}}\right|$$

can be bounded above as

(20) $$e<\frac{1}{\sqrt{N}},$$

which reflects the stochastic convergence rate of the method. However, this estimate does not constitute an a priori deterministic bound; rather, it holds in a statistical sense, typically when the calculations are carried out in series and the final estimate results from appropriate averaging.

#### 2.3.1. First Monte Carlo Model (MCM1)

Based on the finite difference equation corresponding to the first FD model (15), the following relation for the function value $\Phi_{i,j}$ at the central node can be derived. This relation expresses $\Phi_{i,j}$ as a weighted average of its neighboring values, with weights determined by the FD operator

(21) $$\Phi_{i,j}=p_{i-1,j}\Phi_{i-1,j}+p_{i,j+1}\Phi_{i,j+1}+p_{i+1,j}\Phi_{i+1,j}+p_{i,j-1}\Phi_{i,j-1},$$

where the coefficients *p* may be interpreted as the selection probabilities of the next move in the random walk.

(22) $$\begin{array}{c} p_{i-1,j}=-\frac{\left(\eta_{2}-\eta_{1}\right)\delta_{a}\left(x_{1(i,j)}-x_{c}\right)h}{8\left(\eta_{1}+\left(\eta_{2}-\eta_{1}\right)H\left(x_{1(i,j)}-x_{c}\right)\right)}+\frac{1}{4},\\ p_{i+1,j}=\frac{\left(\eta_{2}-\eta_{1}\right)\delta_{a}\left(x_{1(i,j)}-x_{c}\right)h}{8\left(\eta_{1}+\left(\eta_{2}-\eta_{1}\right)H\left(x_{1(i,j)}-x_{c}\right)\right)}+\frac{1}{4},\\ p_{i,j+1}=p_{i,j-1}=\frac{1}{4}. \end{array}$$

It follows directly that

(23) $$p_{i-1,j}+p_{i,j+1}+p_{i+1,j}+p_{i,j-1}=1,$$

which ensures that the choice of one out of the four possible move directions is a certain event, and thus the probabilistic model is properly normalized.

#### 2.3.2. Second Monte Carlo Model (MCM2)

For the second finite difference Formulation (17), the domain is split into two subdomains with an interface $\partial \Omega_{1,2}$. A convenient probabilistic interpretation that is consistent with the conservative FD discretization is to view the random walk as taking place on a weighted graph, where edge weights represent conductances. Therefore, transitions from a node to its four neighbors are chosen with probabilities proportional to the corresponding edge conductances.

Let $x_{i,j}$ be a grid node that belongs to material m∈{1,2} with conductivity $\eta_{m}$. Define the conductance $C_{i\to i\pm 1,j}$ (resp. $C_{i\to i,j\pm 1}$) for the horizontal (resp. vertical) edge leaving $x_{i,j}$ as

(24) $$C_{i\to i\pm 1,j}=\left\{\begin{array}{cc} \eta_{m}, & \mathrm{if}\;\mathrm{the}\;\mathrm{edge}\;\mathrm{is}\;\mathrm{entirely}\;\mathrm{in}\;\Omega_{m},\\ \frac{2\eta_{1}\eta_{2}}{\eta_{1}+\eta_{2}}, & \mathrm{if}\;\mathrm{the}\;\mathrm{edge}\;\mathrm{crosses}\;\partial \Omega_{1,2}, \end{array}\right.\;\;\;C_{i\to i,j\pm 1}=\left\{\begin{array}{cc} \eta_{m}, & \mathrm{if}\;\;\;\mathrm{the}\;\;\;\mathrm{edge}\;\;\;\mathrm{is}\;\;\;\mathrm{entirely}\;\;\;\mathrm{in}\;\;\;\Omega_{m},\\ \frac{2\eta_{1}\eta_{2}}{\eta_{1}+\eta_{2}}, & \mathrm{if}\;\;\;\mathrm{the}\;\;\;\mathrm{edge}\;\;\;\mathrm{crosses}\;\;\;\partial \Omega_{1,2}. \end{array}\right.$$

The harmonic average $2\eta_{1}\eta_{2}/\left(\eta_{1}+\eta_{2}\right)$ on edges that intersect the interface enforces the flux continuity across $\partial \Omega_{1,2}$ in the discrete sense.

With the conductances in (24), the transition probabilities from $x_{i,j}$ to its four neighbors are

(25) $$p_{i-1,j}=\frac{C_{i\to i-1,j}}{S_{i,j}},\;\;\;p_{i+1,j}=\frac{C_{i\to i+1,j}}{S_{i,j}},\;\;\;p_{i,j-1}=\frac{C_{i\to i,j-1}}{S_{i,j}},\;\;\;p_{i,j+1}=\frac{C_{i\to i,j+1}}{S_{i,j}},\;\;\;S_{i,j}=\sum_{\nu \in \{\leftarrow ,\to ,↓,↑\}}C_{i\to \nu }.$$

By construction, $p_{i-1,j}+p_{i+1,j}+p_{i,j-1}+p_{i,j+1}=1$. In homogeneous interior nodes (no edge crosses $\partial \Omega_{1,2}$), Equation (25) reduces to the simple unbiased walk with p=1/4 in each direction. At nodes adjacent to the interface, the probability to cross the interface is increased or decreased according to the relative conductivities through the harmonic-mean conductance, thereby ensuring consistency with the flux continuity condition in (8).

If two overlapping nodes $x_{i,j}^{\left(1\right)}\in \Omega_{1,h}$ and $x_{k,l}^{\left(2\right)}\in \Omega_{2,h}$ lie on $\partial \Omega_{1,2}$, the continuity condition $\Phi \left(x_{i,j}^{\left(1\right)}\right)=\Phi \left(x_{k,l}^{\left(2\right)}\right)$ allows treating them as a single Markov state for the purpose of the random walk. A step “across” the interface uses the cross-interface conductance in (24) and lands on the coincident node of the other submesh. Tangential moves along the interface use within-material conductances. With these rules, the standard Monte Carlo estimator (18) converges, as N→∞, to the finite-difference solution of (17) with harmonic averaging at the interface.

### 2.4. Poisson Problem Formulation

Let $\Omega \subset R^{2}$ be a bounded domain decomposed into two disjoint subdomains $\Omega_{1}$ and $\Omega_{2}$ separated by a smooth interface Γ, such that

(26) $$\Omega =\Omega_{1}\cup \Omega_{2}\cup \Gamma .$$

The thermal conductivity is assumed to be piecewise constant,

(27) $$\eta \left(x\right)=\left\{\begin{array}{cc} \eta_{1}>0, & x\in \Omega_{1},\\ \eta_{2}>0, & x\in \Omega_{2}. \end{array}\right.$$

The governing Poisson problem with volumetric source *f* and mixed (Dirichlet and Neumann) boundary conditions reads

(28) $$\left\{\begin{array}{cc} -\nabla \cdot \left(\eta (x)\nabla \Phi (x)\right)=f\left(x\right), & x\in \Omega ,\\ \Phi \left(x\right)=\Phi_{D}\left(x\right), & x\in \Gamma_{\Phi },\\ \eta \left(x\right)\nabla \Phi \left(x\right)\cdot n\left(x\right)=q_{N}\left(x\right), & x_{i}\in \Gamma_{q},\\ {}[[\Phi ]]=0,\;\;\;[[\eta \nabla \Phi \cdot n]]=0, & x_{i}\in \Gamma , \end{array}\right.$$

where $\partial \Omega =\Gamma_{\Phi }\cup \Gamma_{q}$, with $\Gamma_{\Phi }\cap \Gamma_{q}=\emptyset$. Here, $q_{N}$ denotes the boundary heat flux, $n=\left[n_{1}\;\;\;n_{2}\right]$ is the outward unit normal to ∂Ω, while [[·]] stands for the jump across the interface Γ. The transmission conditions ensure continuity of the potential and balance of the normal fluxes between the two materials.

### 2.5. Meshless Finite Difference Model (MFDM)

In constructing the numerical model, we assume that the domain Ω can be of arbitrary shape. Therefore, we generate an irregular cloud of nodes $\Omega \approx \Omega_{h}$, without any connecting structure such as a regular mesh, finite elements, or mapping constraints. The possibility of deriving discrete equations for (28) with such a discretization is offered by a broad class of meshless methods, in which, by definition, the approximation of the unknown function Φ is carried out solely by means of nodes. Among these approaches is the meshless (generalized) finite difference method, where a local approximation is constructed using the weighted MLS technique. In this method, each point of the domain is associated with a neighborhood of *m* nodal neighbors, selected according to a specified criterion, for example, based on distance or topology. On such a set of nodes, referred to as a star or a difference stencil, a local approximation is generated using a polynomial of the low *p*-th order and the Taylor series expansion of the unknown function Φ with respect to the central point $x_{0}$ of the star. As a result, we obtain the problem of minimizing the local approximation error

(29) $$I={\left(PD_{\Phi }-\Phi \right)}^{T}W\left(PD_{\Phi }-\Phi \right),$$

with respect to the unknown parameters, which are the values of the function and its successive partial derivatives (up to the prescribed order) at the central point:

(30) $$D_{\Phi }=\left[\begin{array}{cccccccc} \Phi & \frac{\partial \Phi }{\partial x_{1}} & \frac{\partial \Phi }{\partial x_{2}} & \frac{\partial^{2}\Phi }{\partial x_{1}^{2}} & \frac{\partial^{2}\Phi }{\partial x_{1}x_{2}} & \frac{\partial^{2}\Phi }{\partial x_{2}^{2}} & \ldots & \frac{\partial^{p}\Phi }{\partial x_{2}^{p}} \end{array}\right].$$

The matrix $P=\left[\frac{h_{1}^{\alpha_{1}}h_{2}^{\alpha_{2}}}{\alpha_{1}!\alpha_{2}!}\right]$, where $\alpha_{1}+\alpha_{2}=p$ contains the values of the basis functions (Taylor monomials) at all *m* nodes of the star, $\Phi =\left[\begin{array}{cccc} \Phi_{1} & \Phi_{2} & \ldots & \Phi_{m} \end{array}\right]$ are the nodal values, and W is the diagonal weight matrix, with weights assigned to each node of the star on its main diagonal. For the construction of difference formulas and for defining transition probabilities in the Monte Carlo approach, singular-type weights are used, decaying with the distance from the central point:

(31) $$\omega \left(x\right)=\frac{1}{{∥x-x_{0}∥}^{p+1}+\varepsilon },$$

where ε denotes the machine precision of the employed software, preventing division by zero at the central point. Due to the singularity of the weight function, the interpolating properties of the approximation at the central point are preserved, even though it is constructed on the basis of redundant information. After minimizing *I* with respect to $D_{\Phi }$, we obtain the difference formula matrix

(32) $$M^{\left(p\right)}={\left(P^{T}WP\right)}^{-1}P^{T}W,$$

whose successive rows correspond to subsequent partial derivatives, e.g.,

(33) $$\nabla \Phi \approx \left[\begin{array}{c} \sum_{j=1}^{m}M_{2,j}^{\left(p\right)}\Phi_{j}\\ \sum_{j=1}^{m}M_{3,j}^{\left(p\right)}\Phi_{j} \end{array}\right],\;\;\;\nabla^{2}\Phi \approx \sum_{j=1}^{m}\left(M_{4,j}^{\left(p\right)}+M_{6,j}^{\left(p\right)}\right)\Phi_{j}.$$

Proceeding analogously to the continuous model CM1 and the discrete model FDM1, one can define continuous approximations of Dirac distributions to represent the discontinuity of the heat conduction coefficient and its derivatives along the interface Γ. Here it is assumed that Γ is discretized as $\Gamma_{h}$, consistent with the prescribed density for $\Omega_{h}$. In this case, there is no need to distinguish two separate node clouds in $\Omega_{1}$ and $\Omega_{2}$, since $\Gamma_{h}\subset \Omega_{h}$. The algebraic system of equations corresponding to (28) can be generated using nodal collocation, i.e., by enforcing the difference equations at the nodes of $\Omega_{h}$, depending on the type of equation:

(34) $$\left\{\begin{array}{cc} D_{1}\left(x_{i(1)},x_{\Gamma (1)}\right)\sum_{j=1}^{m}M_{2,j}^{\left(2\right)}\Phi_{j(i)}+D_{2}\left(x_{i(2)},x_{\Gamma (2)}\right)\sum_{j=1}^{m}M_{3,j}^{\left(2\right)}\Phi_{j(i)}+E\left(x_{i},x_{\Gamma }\right)\sum_{j=1}^{m}\left(M_{4,j}^{\left(2\right)}+M_{6,j}^{\left(2\right)}\right)\Phi_{j(i)}=-f\left(x_{i}\right), & x_{i}\in \Omega_{h},\\ \Phi \left(x_{i}\right)=\Phi_{D}\left(x_{i}\right), & x_{i}\in \Gamma_{\Phi },\\ E\left(x_{i},x_{\Gamma }\right)\sum_{j=1}^{m}\left(M_{2,j}^{\left(1\right)}n_{1}+M_{3,j}^{\left(1\right)}n_{2}\right)\Phi_{j}=q_{N}\left(x_{i}\right), & x_{i}\in \Gamma_{q}. \end{array}\right.$$

where $x_{\Gamma }=\left[x_{\Gamma (1)}\;\;\;x_{\Gamma (2)}\right]$ denotes the node located on $\Gamma_{h}$, closest to node $x_{i}=\left[x_{i(1)}\;\;\;x_{i(2)}\right]$, whereas

(35) $$D_{1}\left(x_{i(1)},x_{\Gamma (1)}\right)=\left(\eta_{2}-\eta_{1}\right)\delta_{a}\left(x_{i(1)}-x_{\Gamma (1)}\right),\;\;\;D_{2}\left(x_{i(2)},x_{\Gamma (2)}\right)=\left(\eta_{2}-\eta_{1}\right)\delta_{a}\left(x_{i(2)}-x_{\Gamma (2)}\right)$$

and

(36) $$E\left(x_{i},x_{\Gamma }\right)=\left(\eta_{1}+\left(\eta_{2}-\eta_{1}\right)H\left(x_{i}-x_{\Gamma }\right)\right)$$

It should be noted that the approximation order p=2 is applied to the internal Poisson equations, whereas p=1 is employed for the natural boundary conditions. In difference-based methods, the approximation order may in principle be higher than the order of the underlying differential equation. However, for the Monte Carlo solution to converge to the correct difference solution, the consistency between these orders is a necessary requirement [46]. This stems from the fact that employing a higher approximation order than the order of the operator may lead to difference coefficients that cannot be interpreted as valid transition probabilities (e.g., due to their negativity). In such a case, the randomized algorithm would no longer reflect the underlying physical process, and the resulting solution would converge to an incorrect limit.

### 2.6. Meshless Monte Carlo Model (MMCM)

The construction of the Monte Carlo algorithm with random walk is straightforward, since it follows directly from (34). Nevertheless, it requires a reformulation of the transition probabilities, previously defined for the MCM1 model in (22), as well as the final estimator (18), which in the more general case must also rely on the nodal notation $\bar{N}$ for unknown solution nodes. This is due to the nonhomogeneity of the Poisson equation and the presence of natural boundary conditions. Accordingly, the transition probabilities from the central node $x_{i}$ to one of the m−1 neighboring nodes $x_{j}$ of the star are given as

(37) $$p_{j}=-\frac{a_{i,j}}{a_{i,1}},\;\;\;x_{i}\in \Omega_{h},\;\;\;p_{j}=-\frac{b_{i,j}}{b_{i,1}},\;\;\;x_{i}\in \Gamma_{q},\;\;\;j=2,3,\ldots ,m,$$

where

(38) $$\left\{\begin{array}{c} a_{i,j}=D_{1}\left(x_{i(1)},x_{\Gamma (1)}\right)M_{2,j}^{\left(2\right)}+D_{2}\left(x_{i(2)},x_{\Gamma (2)}\right)M_{3,j}^{\left(2\right)}+E\left(x_{i},x_{\Gamma }\right)\left(M_{4,j}^{\left(2\right)}+M_{6,j}^{\left(2\right)}\right),\\ b_{i,j}=E\left(x_{i},x_{\Gamma }\right)\left(M_{2,j}^{\left(1\right)}n_{1}+M_{3,j}^{\left(1\right)}n_{2}\right), \end{array}\right.$$

under the assumption that the central node, where the approximation is constructed, is always the first node of the star (j=1).

All the rules of the random walk construction described previously remain valid, except that reaching a boundary node lying on $\Gamma_{q}$ does not terminate the path, since the solution value in this node is unknown. Moreover, these nodes may also serve as starting nodes for the simulation, and subsequent moves from them are governed by the general transition probabilities defined in (37). In practice, this means that hit counters must be stored for all visited nodes, not only for the Dirichlet ones, and each visit to a node on $\Gamma_{q}$ increases its counter while the random walk is continued. Such an extended procedure increases the computational effort, because random paths are typically longer when the proportion of Dirichlet nodes is small. However, it does not deteriorate the stability or convergence of the method, since the Monte Carlo estimator remains an approximation of the underlying difference solution (here realized in the meshless form). Consequently, whenever the meshless MLS formulation provides a correct solution, the Monte Carlo algorithm converges as well. The final Monte Carlo estimator takes the form

(39) $$\Phi_{i}\approx \frac{1}{N}\left(\sum_{x_{j}\in \Gamma_{D}}{\bar{N}}_{i,j}\Phi_{D}\left(x_{j}\right)-\sum_{x_{j}\in \Omega_{h}}{\bar{N}}_{i,j}\frac{f\left(x_{j}\right)}{a_{j,1}}+\sum_{x_{j}\in \Gamma_{N}}{\bar{N}}_{i,j}\frac{q_{N}\left(x_{j}\right)}{b_{j,1}}\right).$$

where ${\bar{N}}_{i,j}$ denotes nodal indication counter at *j*-th node starting from *i*-th node. From a physical perspective, the Monte Carlo estimator (39) can be viewed as the energy balance at node $x_{i}$. The first contribution reflects the direct influence of fixed boundary conditions, which act as “energy reservoirs” driving the solution. The second term captures the effect of internal sources or sinks distributed inside the domain, accumulated along the random trajectory as if the particle were absorbing or releasing energy. The third term represents the interaction with natural boundary conditions, where fluxes correspond to prescribed heat or mass transfer across the boundary. Altogether, the random walk reproduces the physical process of diffusion with sources and boundary interactions, where each particle trajectory provides a microscopic realization of the global conservation law.

### 2.7. Inverse Thermal Problems with Material Discontinuity

The previously analyzed Laplace and Poisson problems, although defined for domains with material discontinuities, were forward, well-posed, and well-conditioned tasks. For a given set of input parameters (geometry, thermal conductivities, thermal loading), these problems admit a unique and stable solution, with only minor sensitivity to small perturbations in the input data.

In contrast, inverse thermal problems are considerably more difficult to analyze. These are ill-posed problems, in which one of the input parameters is unknown and must be identified based on additional assumptions and measured data. While the unknown geometry is typically determined during the design phase, material identification and reconstruction of thermal loading are usually carried out for long-standing existing structures. In such cases, the material properties may be unknown or degraded, or certain physical effects may generate thermal loads that cannot be directly measured.

These scenarios belong to the broad class of problems in Structural Health Monitoring (SHM). The additional data are most often temperature measurements ${\bar{\Phi }}_{i}$ (less frequently heat flux), obtained with a known uniform accuracy ΔΦ at selected $n_{\Phi }$ measurement points ${\bar{x}}_{i}$:

(40) $$\Phi \left({\bar{x}}_{i}\right)={\bar{\Phi }}_{i}\pm \Delta \Phi ,\;\;\;i=1,2,\ldots ,n_{\Phi }.$$

The number and spatial distribution of these measurement points should provide sufficient conditioning of the problem, ensuring that small variations in the measurement data do not lead to large variations in the solution. If this cannot be guaranteed, additional regularization techniques are employed (for example, imposing a prescribed degree of smoothness on the solution).

From a mathematical perspective, such inverse problems are typically formulated as nonlinear constrained optimization tasks.

(41) $$\min_{F}J\left(F\right),\;\;\;J\left(F\right)=\frac{1}{n_{\Phi }}\sum_{i=1}^{n_{\Phi }}{\left(\frac{\Phi \left({\bar{x}}_{i},F\right)-{\bar{\Phi }}_{i}}{\Delta \Phi }\right)}^{2},\;\;\;\left|\Phi \left({\bar{x}}_{i}\right)-{\bar{\Phi }}_{i}\right|\leq \Delta \Phi ,\;\;\;i=1,2,\ldots ,n_{\Phi },$$

where F denotes the vector of decision variables, and J(F) is the objective (target, cost) function measuring the discrepancy between computed $\Phi \left({\bar{x}}_{i},F\right)$ and measured temperatures.

From the viewpoint of optimization theory, the necessary condition for the existence of a local minimum is that the gradient of the objective function vanishes at the solution, i.e.,

(42) $$\nabla_{F}J\left(F^{*}\right)=0.$$

The sufficient condition is that, at the same point, the Hessian matrix of $J_{f}$ with respect to F is positive definite, and the gradient of the objective function vanishes at the solution, i.e.,

(43) $$\nabla_{F}^{2}J\left(F^{*}\right)≻0,$$

which ensures that the stationary point $F^{*}$ corresponds to a local minimum rather than a saddle point or maximum. In addition, the existence of a global minimum can be guaranteed only if the objective function J(F) is convex over the admissible domain. In practice, inverse thermal problems are usually non-convex, which implies the possibility of multiple local minima. For this reason, regularization techniques are often employed not only to stabilize the solution but also to improve the convexity of the optimization landscape, thereby increasing the likelihood of finding a physically meaningful global solution.

Although finite difference algorithms, in their standard forms (FDM1 and FDM2) as well as meshless variants (MFDM), are inherently simple, this simplicity and practical efficiency result from the use of a local formulation and nodal collocation. The local formulation eliminates the need for numerical integration, and nodal collocation ensures that each node contributes exactly one equation, avoiding the assembly of local matrices and vectors. These features provide a practical advantage over traditional FEM in terms of computational simplicity, without implying explicit claims concerning execution time.

In the context of numerical optimization, however, one typically needs to repeatedly solve a series of forward problems for trial values of unknown parameters, as in search methods, conjugate direction methods, gradient-based schemes (steepest descent, conjugate gradients, Newton’s method), or evolutionary strategies (e.g., genetic algorithms). Deterministic methods (FDM, FEM, and others) provide solutions at all nodes of the discretization grid, regardless of the number of measurement points, which is typically much smaller than the number of nodes ($n_{\Phi }\ll n$). Here, Monte Carlo algorithms are particularly useful: due to their point-wise formulation, they can be restricted to only those measurement points where temperature data are required. Furthermore, in the proposed meshless Monte Carlo approach, the relation between temperature and both material and loading parameters is explicit (generally nonlinear, sometimes linear), allowing the gradient of the objective function to be derived analytically. Unknown parameters can then be determined from a relatively small system of algebraic equations, whose size is independent of *n*. This strategy avoids the need for initial guesses, predefined variable bounds, or multiple repeated forward problem solutions, while maintaining convergence and simplicity.

#### 2.7.1. Material Parameter Identification

By substituting the Monte Carlo estimate for the temperature in the meshless model (39) together with the inequality constraints into the objective function (41) and then differentiating it analytically with respect to the decision parameters $F=[\eta_{1}\;\;\;\eta_{2}]$, we obtain two nonlinear equations of the form

(44) $$\left\{\begin{array}{c} \frac{\partial J}{\partial \eta_{1}}=\sum_{i=1}^{n_{\Phi }}\left(\Phi \left({\bar{x}}_{i}\right)-\left({\bar{\Phi }}_{i}\pm \Delta \Phi \right)\right)\frac{\partial \Phi \left({\bar{x}}_{i}\right)}{\partial \eta_{1}}=0\\ \frac{\partial J}{\partial \eta_{2}}=\sum_{l=1}^{n_{\Phi }}\left(\Phi \left({\bar{x}}_{i}\right)-\left({\bar{\Phi }}_{i}\pm \Delta \Phi \right)\right)\frac{\partial \Phi \left({\bar{x}}_{i}\right)}{\partial \eta_{2}}=0 \end{array}\right.$$

where

(45) $$\left\{\begin{array}{c} \frac{\partial \Phi \left({\bar{x}}_{l}\right)}{\partial \eta_{1}}=\frac{1}{N}\left(\sum_{x_{j}\in \Gamma_{D}}\frac{\partial {\bar{N}}_{i,j}}{\partial \eta_{1}}\Phi_{D}\left(x_{j}\right)-\sum_{x_{j}\in \Omega_{h}}f\left(x_{j}\right)\left(\frac{\partial {\bar{N}}_{i,j}}{\partial \eta_{1}}\frac{1}{a_{j,1}}-{\bar{N}}_{i,j}{\left(\frac{\partial a_{j,1}}{\partial \eta_{1}}\right)}^{-2}\right)+\sum_{x_{j}\in \Gamma_{N}}q_{N}\left(x_{j}\right)\left(\frac{\partial {\bar{N}}_{i,j}}{\partial \eta_{1}}\frac{1}{b_{j,1}}-{\bar{N}}_{i,j}{\left(\frac{\partial b_{j,1}}{\partial \eta_{1}}\right)}^{-2}\right)\right)\\ \frac{\partial \Phi \left({\bar{x}}_{l}\right)}{\partial \eta_{2}}=\frac{1}{N}\left(\sum_{x_{j}\in \Gamma_{D}}\frac{\partial {\bar{N}}_{i,j}}{\partial \eta_{2}}\Phi_{D}\left(x_{j}\right)-\sum_{x_{j}\in \Omega_{h}}f\left(x_{j}\right)\left(\frac{\partial {\bar{N}}_{i,j}}{\partial \eta_{2}}\frac{1}{a_{j,1}}-{\bar{N}}_{i,j}{\left(\frac{\partial a_{j,1}}{\partial \eta_{1}}\right)}^{-2}\right)+\sum_{x_{j}\in \Gamma_{N}}q_{N}\left(x_{j}\right)\left(\frac{\partial {\bar{N}}_{i,j}}{\partial \eta_{2}}\frac{1}{b_{j,1}}-{\bar{N}}_{i,j}{\left(\frac{\partial b_{j,1}}{\partial \eta_{2}}\right)}^{-2}\right)\right) \end{array}\right.$$

The derivatives of coefficients $a_{j,1}$ and $b_{j,1}$ with respect to $\eta_{1}$ and $\eta_{2}$ can be obtained without major difficulty by means of analytical symbolic differentiation. A more demanding step is associated with the derivatives with respect to the nodal indication numbers ${\bar{N}}_{i,j}$—these are functions of the material parameters, since the probabilities (37) also depend on them. For this reason, the above system must be solved numerically, similarly to the derivatives $\frac{\partial {\bar{N}}_{i,j}}{\partial \eta_{1}}$ and $\frac{\partial {\bar{N}}_{i,j}}{\partial \eta_{2}}$, which may be computed using appropriate finite difference formulas, where analytical differentiation is replaced with small though finite perturbations, for instance, $\frac{\partial {\bar{N}}_{i,j}}{\partial \eta_{1}}\approx \frac{{\bar{N}}_{i,j}\left(\eta_{1}+\Delta \eta \right)-{\bar{N}}_{i,j}\left(\eta_{1}-\Delta \eta \right)}{2\Delta \eta }$, Δη—sufficiently small (e.g., $10^{-6}$)—which requires performing two Monte Carlo simulations for each derivative.

#### 2.7.2. Recovery of the Heat Source Term

The case of reconstructing loading parameters, most often related to the internal heat source function $f\left(x\right)$, i.e., the right-hand side of the Poisson differential equation, is considerably easier to handle numerically than the identification of material constants. This is due to the fact that the values of this function appear only in a single term of the estimator (39), and its dependence on the temperature at a measurement point is purely linear. The number of parameters $n_{f}$ describing the unknown function must be chosen so that the reconstruction is unique, which means $n_{f}\ll n_{\Phi }$, in order to avoid ill-conditioning of the problem. For illustrative purposes we assume its simplest form—a constant distribution, i.e., $f\left(x\right)=f=\mathrm{const}$. With this assumption, the objective function reduces to a single decision variable $F=\left[f\right]$, and the necessary condition for the existence of a minimum becomes a single linear algebraic equation of the form

(46) $$\frac{dJ}{df}=\sum_{i=1}^{n_{\Phi }}\left(\Phi \left({\bar{x}}_{i}\right)-\left({\bar{\Phi }}_{i}\pm \Delta \Phi \right)\right)\sum_{x_{j}\in \Omega_{h}}\frac{{\bar{N}}_{i,j}}{a_{j,1}}=0,$$

which, after straightforward manipulations, yields an explicit closed-form expression for the unknown loading parameter and simultaneously allows one to determine its accuracy interval:

(47) $$f\pm \Delta f=\frac{\sum_{i=1}^{n_{\Phi }}\left(\sum_{x_{j}\in \Gamma_{D}}{\bar{N}}_{i,j}\Phi_{D}\left(x_{j}\right)+\sum_{x_{j}\in \Gamma_{N}}{\bar{N}}_{i,j}q_{N}\left(x_{j}\right)b_{j,1}^{-1}-N\cdot \left({\bar{\Phi }}_{i}\pm \Delta \Phi \right)\right)\sum_{x_{j}\in \Omega_{h}}{\bar{N}}_{i,j}a_{j,1}^{-1}}{\sum_{i=1}^{n_{\Phi }}{\left(\sum_{x_{j}\in \Omega_{h}}{\bar{N}}_{i,j}a_{j,1}^{-1}\right)}^{2}}.$$

The entire solution procedure requires only a single Monte Carlo simulation carried out at all $n_{\Phi }$ temperature measurement points.

## 3. Results

The developed algorithms for solving the thermal problem with a material discontinuity were tested on a variety of benchmark cases of increasing complexity, starting from the Laplace problem on a regular grid, through a direct problem (with prescribed material parameters and a known source function), up to the Poisson problem, geometrically complex domains, and ill-posed inverse problems. Representative results are presented in this section. The main attention is focused on such aspects of the algorithms as the accuracy of the obtained temperature field with respect to the prescribed analytical solution (for difference-based methods underlying the FDM1 and FDM2 models as well as the mesh-free MFDM model) or with respect to the reference difference solution (for Monte Carlo-based approaches, i.e., the MCM1, MCM2, and MMCM models). In addition, the sensitivity of the algorithms to model parameters such as the discretization density (difference methods), the number of random paths (Monte Carlo methods), and the assumed measurement accuracy (inverse problems) is also investigated.

### 3.1. Direct Problem for the Laplace Equation on a Rectangular Domain

In the first example, we consider a square domain of unit size and the Laplace equation formulated in (1), with a vertical interface located at $x_{c}=0.5$ separating two rectangular subdomains with $\eta_{1}=1$ and $\eta_{2}=100$. A non-trivial reference solution is assumed in the form

(48) $$\Phi \left(x_{1},x_{2}\right)=\left\{\begin{array}{cc} \eta_{2}\sinh\left(k(x_{1}-x_{c})\right)\cos\left(kx_{2}\right), & x_{1}<x_{c},\\ \eta_{1}\sinh\left(k(x_{1}-x_{c})\right)\cos\left(kx_{2}\right), & x_{1}\geq x_{c}, \end{array}\right.$$

which satisfies the Laplace equation and serves as the basis for prescribing the Dirichlet boundary conditions introduced in (2).

From a physical point of view, this solution represents a stationary temperature distribution in a two-layer composite medium, where the vertical interface models the contact between two strongly contrasting materials. The conductivity coefficients $n_{1}$ and $n_{2}$, differing by two orders of magnitude, can be interpreted as a thermally insulating layer attached to a highly conductive substrate (for instance, air–metal or ceramic–steel). The functional form involving hyperbolic sine and cosine ensures both the fulfillment of the Laplace equation and the continuity of heat fluxes across the interface. This benchmark configuration provides a stringent test for the stability and accuracy of the proposed algorithms under conditions of a sharp material discontinuity.

The solution of the problem was obtained for four models, two deterministic (FDM1 and FDM2) and two stochastic (MCM1 and MCM2). For FDM1 and MCM1, the approximation parameter of the Dirac delta function (12) was set to a=h/2, where *h* denotes the mesh size in the square domain for the assumed number of nodes along the boundary. Stars with m=16 nodes were generated at each internal node.

**Figure 1:**
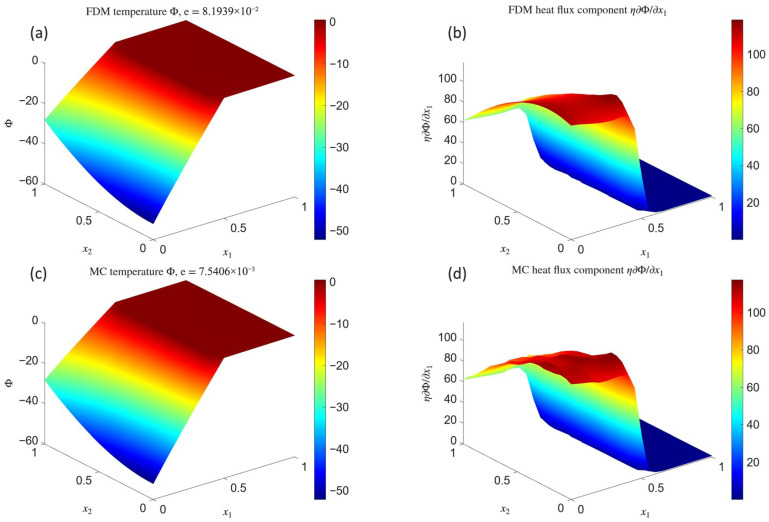
Laplace problem solution: distributions of the temperature field Φ (**a**,**c**) and the heat flux component $\eta \frac{\partial \Phi }{\partial x_{1}}$ (**b**,**d**) obtained using FDM1 (**a**,**b**) and MCM1 (**c**,**d**).

In the first step, the analysis of numerical solutions obtained for the first continuous model (CM1), in which the discontinuity is represented by the distribution, was carried out. Figure 1 presents the spatial distributions of the temperature field Φ and the heat flux component $\eta \frac{\partial \Phi }{\partial x_{1}}$, where a jump-type discontinuity occurs. The results were obtained for a regular mesh with 21×21=441 nodes (with mesh size h=0.05). The upper row shows the results obtained using FDM1, while the lower row corresponds to MCM1 with N=5000 random paths. The titles of the temperature plots report the relative global errors of the solution in the $L_{2}$ norm, with respect to the reference solution (48) (for FDM1) and the finite difference solution (for MCM1).

**Figure 2:**
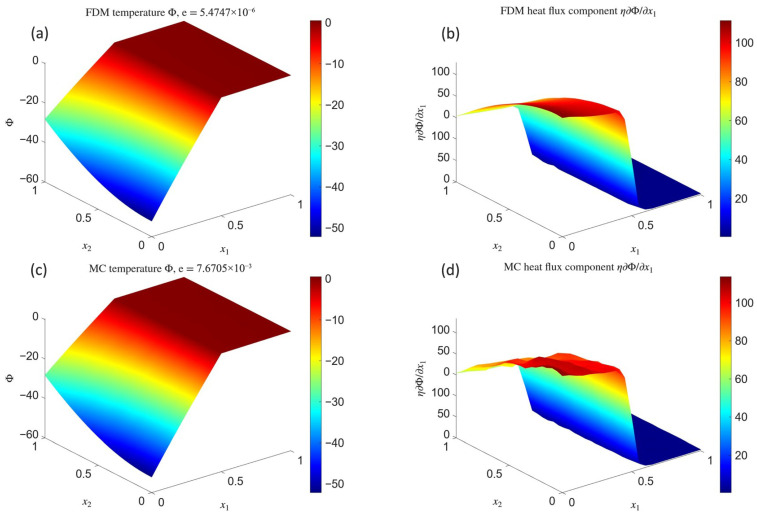
Laplace problem solution: distributions of the temperature field Φ (**a**,**c**) and the heat flux component $\eta \frac{\partial \Phi }{\partial x_{1}}$ (**b**,**d**) obtained using FDM2 (**a**,**b**) and MCM2 (**c**,**d**).

A similar comparison was carried out for the second continuous model (CM2), in which the discontinuity is introduced through interface conditions imposed on the temperature and heat flux. Accordingly, Figure 2 gathers the distributions of the temperature and the discontinuous heat flux component obtained with FDM2 and MCM2.

**Figure 3:**
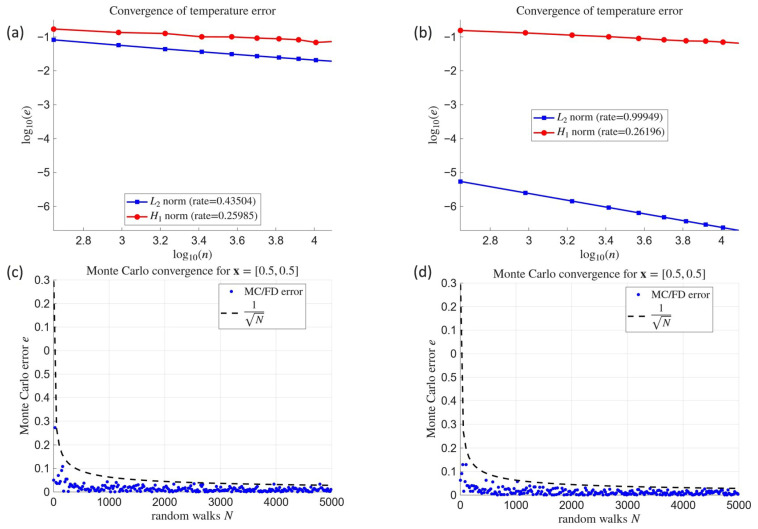
Convergence analysis: *h*-convergence results for FDM1 (**a**) and FDM2 (**b**) and *N*-convergence results for MCM1 (**c**) and MCM2 (**d**).

Subsequently, convergence tests typical of each model were performed. For the deterministic models FDM1 and FDM2, *h*-convergence was examined by systematically refining the mesh density, thus generating a sequence of regular grids from a coarse mesh with 21×21=441 nodes up to a very fine mesh with 111×111=12,321 nodes. For each grid, an error estimator for the temperature field was computed with respect to the reference solution (48), using the $L_{2}$ norm (blue squares) and the $H_{1}$ semi-norm (red circles). The corresponding results are presented in the upper row of Figure 3 for FDM1 and FDM2, respectively (the same scale for both graphs was used), in a full logarithmic scale. The estimated convergence rates, computed as the slopes of linear regression lines fitted to the sets of points obtained for successive mesh refinements and displayed in legends, quantify the rate of error decay.

Similarly, for the stochastic models MCM1 and MCM2, the convergence of the solution at a selected interface point $x=\left[0.5\;\;\;0.5\right]$ (where a jump in material properties occurs) was investigated as a function of the number of random paths, varied from N=5 to N=5000 with a step of 20. For reference, the theoretical error bound (20) is also plotted.

### 3.2. Direct Problem for the Poisson Equation on Building-Shaped Domain with Material Interface

**Figure 4:**
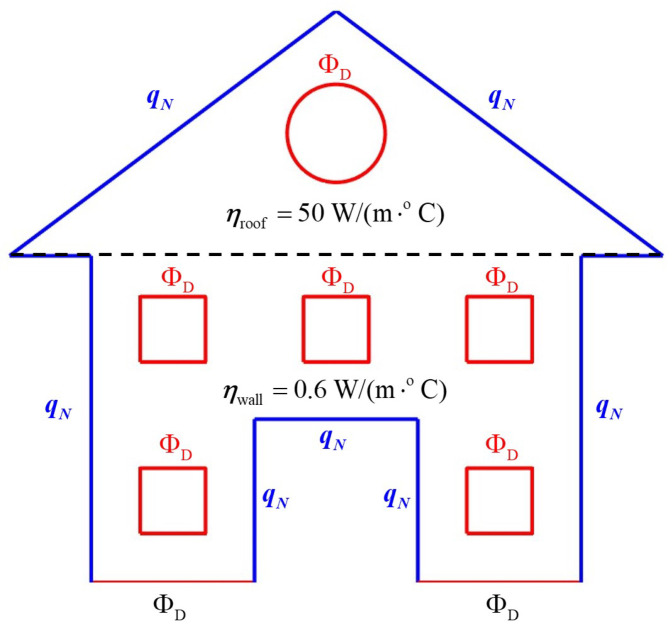
Schematic diagram of the building-shaped domain with the assigned material properties and boundary conditions (blue denotes the natural (Neumann) boundary conditions for the heat flux, while red denotes the essential (Dirichlet) boundary conditions for the temperature).

In order to assess the performance of the proposed meshless FD and Monte Carlo approaches on a more engineering-oriented problem, we consider a two-dimensional domain in the shape of a house with doors and windows. The geometry is divided into two subdomains separated by an interface: the front wall and the roof (Figure 4). To emphasize the discontinuity in the vertical component of the heat flux, we adopt the most contrasting variant, where the thermal conductivities of the two materials differ by an order of magnitude. Specifically, we assume

(49) $$\eta_{\mathrm{wall}}=0.6\;W/\left(m\cdot {}^{\circ}C\right),\;\;\;\;\;\;\eta_{\mathrm{roof}}=50\;W/\left(m\cdot {}^{\circ}C\right).$$

The value $\eta_{\mathrm{wall}}=0.6$ corresponds to a low–conductivity building material, such as porous brick or mineral wool insulation, whereas $\eta_{\mathrm{roof}}=50$ is representative of highly conductive materials, such as steel, aluminium, or crystalline stone (e.g., granite). This strong contrast ensures a pronounced discontinuity in the heat flux across the interface.

As a reference solution, we prescribe the trigonometric temperature distribution

(50) $$\Phi \left(x\right)=10\left(\sin\left(x_{1}\right)+\cos\left(x_{2}\right)\right),$$

expressed in degrees Celsius. Physically, this profile can be interpreted as a standing thermal wave, producing alternating hot and cold regions in the domain. Such a sinusoidal distribution may be regarded as a simplified model of periodic thermal loading, for instance due to cyclic solar radiation or diurnal heating patterns. The oscillatory form ensures a nontrivial distribution of both temperature and heat flux, which is particularly useful for validating numerical methods.

Concerning boundary conditions, the building is assumed to be thermally insulated from the ground and through window openings, which corresponds to Dirichlet conditions. Neumann conditions, prescribing the heat flux, are imposed on all external wall and roof boundaries.

This setup, combining realistic material contrast with a smooth analytical solution, provides a stringent benchmark for evaluating the accuracy of the worked-out solvers in domains with internal interfaces.

**Figure 5:**
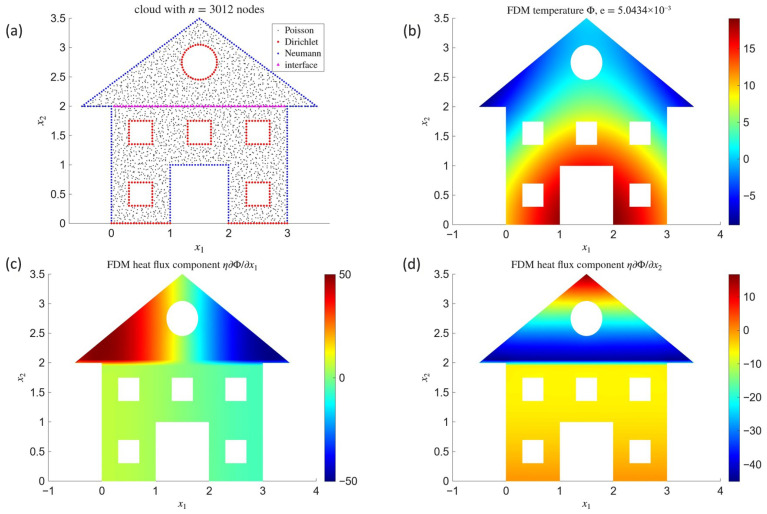
Cloud of 3012 nodes (**a**) with results obtained by means of MFDM: temperature (**b**) and components of heat flux (**c**,**d**).

Figure 5 shows the numerical results obtained with the MFDM model. Graph on Figure 5a illustrates the cloud of 3012 nodes fitted to the geometry of the considered domain. All interior nodes were additionally perturbed randomly with an amplitude of up to 30% of their initial positions. The graphs in Figure 5b present the temperature field, while Figure 5c,d depict the components of the heat flux vector.

**Figure 6:**
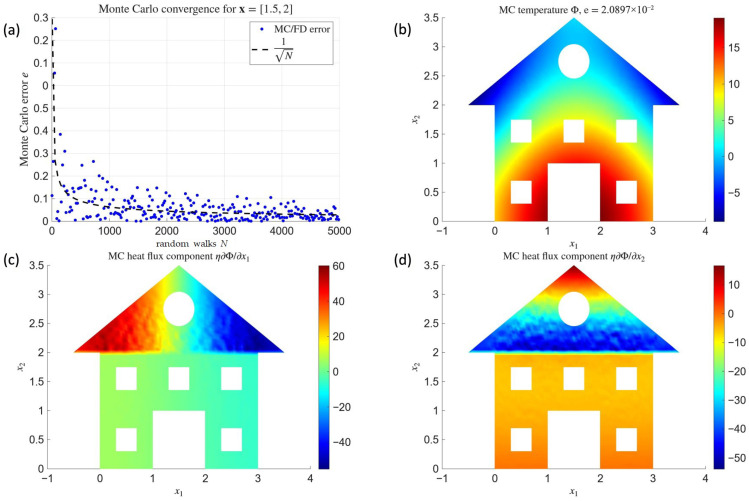
Results obtained by means of MMCM: *N*-convergence of one selected value (**a**), temperature (**b**), and heat flux components (**c**,**d**).

Figure 6 shows the results obtained with the MMCM model (mesh-free Monte Carlo method), which provides a stochastic estimation of the MFDM solution. The graph in Figure 6a presents the convergence of the Monte Carlo solution toward the reference finite-difference solution at a selected interface point, located at x=[1.5,   2]m, as a function of the number of random paths *N*. The graphs in Figure 6b–d display, respectively, the temperature distribution and the two components of the heat flux. As in the previous case, pointwise values are shown without any additional smoothing.

### 3.3. Inverse Problem for the Poisson Equation on Building-Shaped Domain with Material Interface

The last series of tests was carried out for the inverse Poisson problem in a building-shaped domain, in which a single unknown parameter of the internal heat source *f* had to be identified. The same thermal conductivity coefficients were applied for the walls and roof as in (49). This time, however, the boundary conditions and the load function were chosen in such a way that they did not correspond to any analytical solution. In this manner, realistic conditions were simulated: zero temperature at the locations where the building touches the ground (contact with the substrate), zero flux on the building walls, and a positive heat flux $q_{N}=10\;W/m^{2}$ on the roof edges, modeling solar irradiation.

In order to generate simulated temperature measurements, the problem was solved numerically using the MFDM, assuming a single-parameter internal heat source $f=10\;W/m^{3}$. This simplified assumption corresponds to a spatially uniform volumetric generation rate, which can be interpreted as the average value of a weakly varying source term inside the domain. Since the actual distribution is not known a priori, such a representation allows one to at least identify the correct order of magnitude of the heat source from the inverse analysis. The obtained temperature measurements were additionally perturbed with a prescribed amplitude ΔΦ (also representing measurement accuracy), while the measurement points were selected so as to cover the entire computational domain.

**Figure 7:**
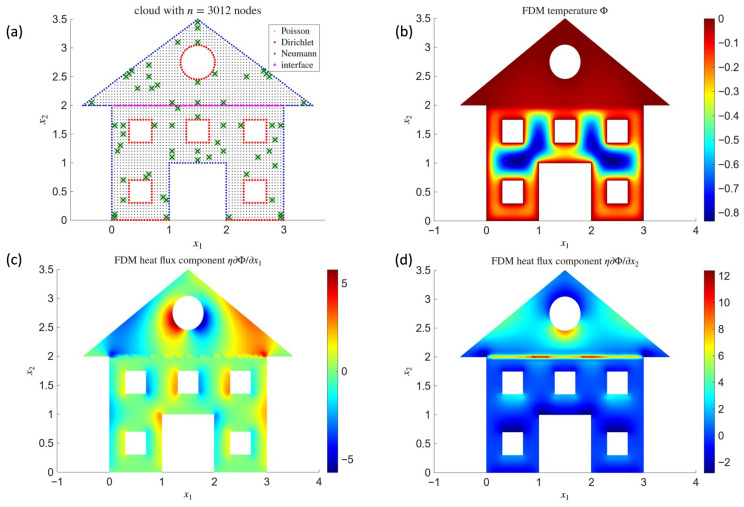
Reconstruction of the internal heat source: adopted discretization and distribution of measurement points (green cross marks) (**a**) and plots of the temperature (**b**) and heat flux components (**c**,**d**) after full reconstruction.

An exemplary distribution of $n_{\Phi }=61$ measurement points against the discretization with n=3012 nodes is shown in Figure 7a. The remaining plots in Figure 7b–d present the reconstructed temperature field and the heat flux components obtained after solving the inverse problem with the Monte Carlo method, i.e., by applying Formula (47), which yielded the stochastic estimation of the optimal solution $f_{MC}=10.21\;W/m^{3}\pm 1.2\;W/m^{3}$, for N=100 and ΔΦ=0.1 °C.

**Figure 8:**
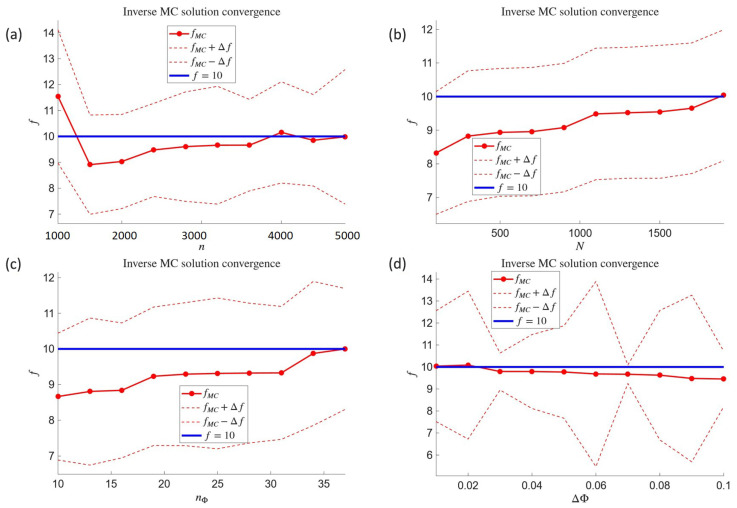
Convergence tests for the inverse problem of internal heat source reconstruction: *n*-convergence (**a**), *N*-convergence (**b**), $n_{\Phi }$-convergence (**c**), and ΔΦ-convergence (**d**). The two red dashed lines correspond to $f_{\mathrm{MC}}+\Delta f$ (upper line) and $f_{\mathrm{MC}}-\Delta f$ (lower line).

Next, the influence of four parameters on the accuracy of the inverse problem solution was investigated. In each of the four tests, one parameter was varied while the others were kept fixed at the following levels: n=3012, N=1000, $n_{\Phi }=21$, and ΔΦ=0.1 °C. In the first test, the discretization density was varied from n=1000 to n=5000. In the second, the number of random paths was varied from N=100 to N=2000. The third test examined the number of temperature measurement points, varied from $n_{\Phi }=10$ to $n_{\Phi }=36$. The fourth test assumed variation of the measurement accuracy from ΔΦ=0.01 °C to ΔΦ=0.1 °C. The results of all four tests are shown in the subsequent subplots of Figure 8—for each case, the convergence of the solution $f_{MC}$ and its error bounds are presented, along with the reference value $f=10\;W/m^{3}$, based on which the simulated measurements were generated.

All results presented in this work were obtained using a prototype software developed by the authors in Matlab 2022b, executed on an Asus laptop equipped with an Intel(R) Core(TM) CPU 1.80 GHz and 16 GB of RAM. Computation times are not reported, as they were negligible due to the simplicity of the analyzed problems. The only exception was the application of the MCM2 model to the Laplace problem in a rectangular domain. Owing to the complexity of the numerical model, solving the task with the Monte Carlo method for all mesh points took approximately one minute, whereas all other computations required no more than a few to several seconds.

## 4. Discussion

The obtained results fully confirm the correctness of the developed algorithms and their applicability to the numerical analysis of problems with jump-type material discontinuities. It should be emphasized that these algorithms are based on relatively simple mathematical and numerical models, relying on local formulations of the problem (without global domain integration or the introduction of additional unknowns in the form of variational terms) and on difference-based methods, where the central role is played by the node rather than the subdomain. This node-centered perspective makes it easier and more natural to capture sharp changes in solution derivatives across material interfaces, in contrast to element-based approaches. From the materials-science perspective, such an ability is of particular importance in the modeling of composite structures, layered ceramics, or thermal barrier coatings, where abrupt jumps in thermal conductivity or elastic modulus are the rule rather than the exception.

The Laplace test models on a rectangular domain demonstrate that deterministic solutions can be obtained in two alternative ways. The first approach, corresponding to models FC1 and FDM1, is easier to implement, and both the solution accuracy (Figure 1a) and its $L_{2}$-convergence rate (Figure 3a) are satisfactory ($e∼10^{-2}$). The second approach, represented by FC2 and FDM2, requires more implementation effort, yet the accuracy is clearly superior ($e∼10^{-6}$, Figure 2a), and the convergence rate (Figure 3b) nearly coincides with the theoretical estimate $e<Ch^{p-k+1}$, where k=2 is the order of the differential equation and *C* is a constant independent of *h* and *p*. The semi-norm $H_{1}$, which essentially measures the accuracy of the solution gradient, exhibits large values and slow convergence in both cases. This can be explained by the fact that $H_{1}$ was computed globally by means of central finite difference schemes, thus with averaging across the material interface, whereas the reference gradient of solution (48) is discontinuous at this location. In physical terms, this reflects the difficulty of capturing steep thermal flux gradients across heterogeneous media where conductivity changes abruptly. It is worth noting that the plots of the horizontal heat flux component (Figure 1b and Figure 2b) accurately reproduce this discontinuity in both models, even though the underlying node grid was relatively coarse. Such robustness suggests a promising potential for simulating real-world layered or composite materials where capturing flux mismatches is crucial for predicting service performance.

The stochastic Monte Carlo solutions, corresponding to models MCM1 and MCM2, are fully comparable to their difference-based counterparts. Moreover, when comparing the temperature plots (Figure 1a vs. Figure 1c and Figure 2a vs. Figure 2c), it is difficult to notice any significant differences despite the fact that the Monte Carlo solution was obtained point by point. Due to its stochastic nature, maintaining uniform accuracy between points was not possible, yet the low error relative to the deterministic solution indicates that the Monte Carlo approach can serve as a viable surrogate—if not over the entire mesh, then at least in selected locations of interest. This may be particularly advantageous in multiscale analyses of heterogeneous materials, where detailed local resolution is required only in subregions with strong gradients, such as interfaces between fibers and matrix in composites. The random character of the temperature field is more apparent in the plots of the discontinuous flux component (Figure 1d and Figure 2d), where pronounced oscillations in high-value regions reveal the low degree of temperature smoothness. While additional numerical smoothing could be applied, it was intentionally avoided here to better illustrate the raw character of such solutions. Importantly, the Monte Carlo solutions converge towards their deterministic counterparts as the number of random paths increases, although this convergence is stochastic, non-uniform, and may locally deviate from theoretical estimates, as demonstrated in Figure 3c (MCM1) and Figure 3d (MCM2). In practical terms, such stochastic convergence mirrors the statistical variability of transport phenomena in heterogeneous media, where microscale fluctuations coexist with macroscale averages.

The Monte Carlo error in Figure 6a follows a hyperbolic decay trend with increasing *N*, as expected for stochastic methods. However, the convergence is less strict compared to the Laplace equation solved on a rectangular domain, due to the higher complexity of the present geometry with material interface and heterogeneous conductivity. The temperature and flux components obtained with the MMCM (Figure 6b–d) reproduce the qualitative behavior of the MFDM results (Figure 5b–d), though they exhibit local variability in smoothness depending on the spatial location. This feature reflects the stochastic nature of the method and highlights the trade-off between accuracy and computational cost in Monte Carlo simulations.

The results obtained for the inverse Poisson problem in the form of reconstructing a single-parameter heat source, presented in Figure 7 as outcomes for fixed parameter values and in Figure 8 as convergence test results, confirm the correct performance of Formula (47). It is worth emphasizing that the method is self-starting; i.e., the estimation of the heat source parameter and its error bounds does not require any prior knowledge of its order of magnitude. All necessary quantities are obtained in exactly the same manner as for the directly formulated well-posed problem. Moreover, the number of measurement points is of lesser importance in this case, provided that their distribution is adequately adapted to the geometry of the domain.

## 5. Conclusions

The main objective of this study was to develop and test standard and meshless Monte Carlo-based frameworks for solving forward and inverse problems of steady-state heat conduction with a discontinuous conductivity. The methodology was implemented in a prototype Matlab code and applied to a series of benchmark cases of increasing complexity. Particular attention was given to the inverse formulation, where the task consisted of reconstructing unknown parameters from temperature measurements.

In the direct problems, the efficiency and accuracy of the proposed meshless approach were verified on simple geometries and boundary conditions, including a two-layered material domain. For the inverse problem, two classes of tasks were considered: identification of material parameters and reconstruction of a heat source term. The latter was analyzed in detail, with special focus on the single-parameter case, where a closed-form solution formula was derived and validated. The obtained results confirmed both the correctness and the robustness of the proposed method. Importantly, the inverse procedure is fully self-starting and does not require any prior knowledge of the magnitude of the sought parameters.

The scope of the present work was intentionally limited to relatively simple settings, which nevertheless allowed for a transparent illustration of the potential of the approach. Several promising directions for further research can be outlined. These include the extension of the inverse identification to multiple material parameters, multi-parameter heat sources, and multilayered materials beyond the two-layer case considered here. Another important avenue is the application of the method to fully three-dimensional geometries and to coupled thermo-mechanical problems. A further challenge is the occurrence of air gaps due to differential shrinkage of adjacent materials at elevated temperatures. This leads to evolving computational domains, where mesh-free methods may offer particular advantages over the FEM by avoiding costly remeshing. Another promising extension is to account for temperature-dependent thermal conductivities, which would introduce nonlinearity into the formulation and require iterative solution strategies, for example, based on Newton–Raphson updates of transition probabilities in the random walk scheme. These extensions will allow the proposed framework to address a broader range of practical engineering applications.

## Data Availability

The original contributions presented in this study are included in the article. Further inquiries can be directed to the author.
